# Accuracy and Reproducibility of Adipose Tissue Measurements in Young Infants by Whole Body Magnetic Resonance Imaging

**DOI:** 10.1371/journal.pone.0117127

**Published:** 2015-02-23

**Authors:** Jan Stefan Bauer, Peter Benjamin Noël, Christiane Vollhardt, Daniela Much, Saliha Degirmenci, Stefanie Brunner, Ernst Josef Rummeny, Hans Hauner

**Affiliations:** 1 Department of Neuroradiology, Technische Universität München, Munich, Germany; 2 Department of Radiology, Technische Universität München, Munich, Germany; 3 Else Kröner-Fresenius-Center for Nutritional Medicine, Technische Universität München, Munich, Germany; University of Ulm, GERMANY

## Abstract

**Purpose:**

MR might be well suited to obtain reproducible and accurate measures of fat tissues in infants. This study evaluates MR-measurements of adipose tissue in young infants *in vitro* and *in vivo*.

**Material and Methods:**

MR images of ten phantoms simulating subcutaneous fat of an infant’s torso were obtained using a 1.5T MR scanner with and without simulated breathing. Scans consisted of a cartesian water-suppression turbo spin echo (wsTSE) sequence, and a PROPELLER wsTSE sequence. Fat volume was quantified directly and by MR imaging using k-means clustering and threshold-based segmentation procedures to calculate accuracy *in vitro*. Whole body MR was obtained in sleeping young infants (average age 67±30 days). This study was approved by the local review board. All parents gave written informed consent. To obtain reproducibility *in vivo*, cartesian and PROPELLER wsTSE sequences were repeated in seven and four young infants, respectively. Overall, 21 repetitions were performed for the cartesian sequence and 13 repetitions for the PROPELLER sequence.

**Results:**

*In vitro* accuracy errors depended on the chosen segmentation procedure, ranging from 5.4% to 76%, while the sequence showed no significant influence. Artificial breathing increased the minimal accuracy error to 9.1%. *In vivo* reproducibility errors for total fat volume of the sleeping infants ranged from 2.6% to 3.4%. Neither segmentation nor sequence significantly influenced reproducibility.

**Conclusion:**

With both cartesian and PROPELLER sequences an accurate and reproducible measure of body fat was achieved. Adequate segmentation was mandatory for high accuracy.

## Introduction

Mean birth weight and body fat mass growth in the first year of life have increased continuously over the last decades [1,2]. Both elevated birth weight and early fat mass are risk factors for childhood and adult obesity [3,4]. Compared with 1985 and 1999 there is a 50% increase in overweight children and adolescents today [5]. To estimate the risk of associated diseases in later life, such as type 2 diabetes, not only the total amount of fat but also the distribution of body fat is of major importance [6]. However, up to now very little is known about the development of different adipose tissues in young infants [7–9].

To advance this knowledge, reproducible measures are needed to evaluate body fat non-invasively in observational studies that try to find early predictors for obesity and diabetes in later life. MR has shown to be well suited for this task, in particular with recent developments in sequence design [9–11]. However, it might be a challenge to obtain accurate and reproducible quantitative measures in young infants, as motion artifacts have to be minimized and breathing artifacts cannot completely be avoided. The peculiarities of this age group entail high demands in sequence design and post processing algorithms. In particular, the segmentation procedure is a demanding process, that possibly leads to high reproducibility and accuracy errors. Different segmentation algorithms have been compared in adults, however results will change in an infant’s anatomy [12]. To our knowledge, neither data about accuracy nor reproducibility of measures of adipose tissue in this age group are available in the literature.

Thus, the first purpose of this study was to evaluate the accuracy of adipose tissue measurements in phantoms by MRI, using water displacement as the reference standard. Based on these results, the second purpose was to evaluate the feasibility and reproducibility of adipose tissue measurements in infants and to compare the influence of different MRI sequences and segmentation algorithms."

## Material and Methods

This prospective study was approved by the local institutional review board (IRB of the Klinikum rechts der Isar, Technische Universität München) and conducted according to the principles expressed in the Declaration of Helsinki. All parents gave written informed consent. The study was designed to evaluate the quantification of adipose tissue in young infants by whole body MR. Accuracy was assessed *in vitro* in ten phantoms and reproducibility *in vivo* in ten young infants. The analysis of MRI data results in the expression of adipose tissue content as volume of adipose tissue, and was reported as volume in the present study. However, a conversion to adipose tissue mass is possible on the assumption that the density of adipose tissue is 0.925g/cm^3^ [13,14].

## Phantom studies

To determine accuracy of MR-based fat measurements *in vitro*, a phantom was build, simulating an infant’s torso. It consisted of two plastic tubes filled with water and a total diameter of about 15cm. A third, empty tube was placed between the two water-filled tubes and connected to a respiration machine to simulate breathing motion with a frequency of 50/min and a volume of 20ml per breath. Different layers of lard with a thickness of 2–4mm each and a width of about 5cm were wrapped around the water tubes. Ten different phantoms were built in this way, with total fat volumes ranging from 54 to 300ml. While the geometrical dimensions of the phantom were quite similar to a young infant, the surface-to-volume ratio was much higher in the phantom due to the multiple thin layers of fat. This setup should enhance errors due to incorrect segmentation of partial volume effects.

Fat volumes were quantified directly by putting the bands of lard in a water bath and measuring the replaced water volume (Archimedes method). These measurements were repeated three times and average values were used for all further calculations. The estimated reproducibility error for this method was 1.4%, determined as the root mean square error of the coefficients of variation of each band of lard. The lard was kept in sealed plastic bags to prevent from drying and all other measurements were performed within one week. These volumes determined by the Archimedes method were used as a standard of reference for all image-based calculations. MR images of all ten phantoms were obtained using a 1.5T MR scanner (Avanto, Siemens, Erlangen, Germany) with and without simulated breathing. Scans consisted of a conventional cartesian intermediate-weighted turbo spin echo (TSE) sequence with water suppression (ws), and a Periodically Rotated Overlapping ParallEL Lines with Enhanced Reconstruction (PROPELLER) wsTSE sequence as described below.

Fat volume was calculated in the MR images using an semiautomatic k-means clustering as well as a threshold-based segmentation with varying thresholds.

## Infant studies

To determine reproducibility of the MR-based fat measurements *in vivo*, repeated measurements were obtained in young infants. Previous *in vitro* experiments were used to exclude insufficient protocols from the *in vivo* part of the study. The infants (average age 67days, range 22–154 days) were recruited from a large randomized controlled trial, initially designed to investigate the impact of nutritional fatty acids on adipose tissue development in the offspring. 208 healthy pregnant women were enrolled in the study. Primary outcome was infant fat mass assessed by skinfold thickness measurements up to the first year of life. The study design as well as the clinical results on infant fat mass up to 1 year of age were previously described in detail [15,16]. 188 infants were clinically examined at birth and 170 were followed-up until 1 year post partum. Out of all those infants, 53 additionally underwent MR imaging. In all other infants no appointment could be made for the scan due to time constraints or disagreement of the parents. The infants undergoing MRI were placed in a plastic tray and had to wear ear plugs during the scan. If the infants were not able to fall into natural sleep without sedative drugs within two hours, the procedure was aborted. In 36 infants, MR scans could be performed during natural sleep without motion artifacts. In ten of these infants both cartesian and PROPELLER sequences were acquired and at least one sequence was repeated three or four times for reproducibility measurements. The data obtained in these infants was evaluated in this study. As the complete scan time was limited, infants, falling asleep faster, were scanned with more repetitions. The cartesian wsTSE sequence was repeated three times in seven infants with an average age of 77 days (range 46–124), resulting in 21 scans with a degree of freedom [df = n(infants)*(n(scans per infant) -1)] of 14. The PROPELLER wsTSE sequence was repeated four times in one infant and three times in three infants with an average age of 54 days (range 49–60), resulting in 13 scans with a df = 9. It is to note that in one infant both sequences were acquired three times each; this infant was included in both groups described above.

## MR imaging protocol

All scans were performed in ‘silent mode’ with slow gradients. As suggested by Peng et al. we used water-saturated magnetic resonance imaging for accurate abdominal fat quantification [17]. Protocols were initially set up for a HASTE (Half Fourier Acquisition Single Shot Turbo Spin Echo) sequence, a fast 3D gradient-recalled-echo Dixon sequence, a conventional cartesian wsTSE sequence and a PROPELLER wsTSE sequence to minimize breathing artifacts. The conventional cartesian TSE sequence was a 2D intermediate weighted TSE sequence with a water suppression pulse (Table 1). TE was 14ms, TR 2416ms, the echo train length 5 and the bandwidth 130 Hz/px. Parallel imaging was used with the GRAPPA (GeneRalized Autocalibrating Partially Parallel Acquisition) algorithm and an acceleration factor of R = 2, thus 26x34x60 cm^3^ were covered with a spatial resolution of 0.9 x 0.9 x 5 mm^3^ in 4:56 min. Spacing between the slices was 1mm. In case of the three older infants (> 65 days), the spacing was increased to 2mm, as a scan length of 70cm was needed to cover the whole bodies.

**Table 1:** *Scan parameters of the wsTSE sequences used* (prop: PROPELLER; cart: cartesian).

	cart	prop
TE [ms]	14	47
TR [ms]	2416	2770
scan time [min:sec]	4:56	7:24
Voxel size [mm]	0.9 x 0.9 x 5	1.1 x 1.1 x 5
Slice gap [mm]	1	1
Field of view [cm]	26x34x60	35x35x60

In case of the PROPELLER wsTSE sequence a quadratic field of view has to be used; thus, parameters were adopted to achieve a compromise between resolution and imaging time: TE was 47ms and TR 2770ms, echo train length was 9 and the bandwidth 345 Hz/px. Parallel imaging was used with the GRAPPA algorithm and R = 2, thus 35x35x60 cm^3^ were covered with a spatial resolution of 1.1 x 1.1 x 5 mm^3^ in 7:24 min. Spacing between the slices was 1mm.

In seven infants (out of all 53), we tried to obtain a HASTE sequence. However, due to the inconsistent noise pattern of this sequence, all infants awoke during that scan. Therefore no data about this sequence is included in the presented work. The same applied for a fast 3D gradient-recalled-echo Dixon sequence; scanning of this sequence was aborted after three infants awoke during the scan.

## Segmentation

MR images were anonymized and transferred to an offline workstation. The whole body of the infants was segmented, including head and extremities. It was performed with two different algorithms that were developed in-house and implemented as plugins in ImageJ. [Rasband, W.S., ImageJ, U. S. National Institutes of Health, Bethesda, Maryland, USA, http://rsb.info.nih.gov/ij/, 1997–2009.] These plug-ins combined our C++/ITK based algorithms with the countless options of ImageJ. We applied a threshold-based segmentation, that selects all pixels with a signal intensity higher than a specific threshold. The threshold was defined according to the histogram of the measurements, where a maximum was found at an intensity of about 15 (corresponding to background noise) and of about 420 (corresponding to fat). To separate these two phases, three different thresholds between those peaks (150, 250 and 350) were defined and kept constant for further evaluation. In a second step, all segmented areas with a volume smaller than 25mm^3^ were excluded. In a third step, the user could correct artifacts due to breathing and inhomogeneities of the magnetic field manually.

Additionally, we applied an optimized semi-automatic k-means clustering segmentation algorithm, that is not dependent on a specific threshold. It has been described in detail before [18]. In brief, it performs the following steps for segmentation of MRT data: (i) bias-field effect correction, (ii) parameter estimation for k-means clustering, (iii) k-means clustering and (iv) detection of adipose tissue via active contours. Finally, the user could correct artifacts manually. This semi-automatic segmentation was much faster as compared to the threshold-based segmentation due to fewer user-interaction needed, in case of 100 slices about 15 minutes vs. 30 minutes.

In case of the k-means clustering segmentation we semi-automatically separated subcutaneous from intra-abdominal fat, including intraperitoneal and retroperitoneal fat.

## Statistical analysis

The level of significance was set to be at p < 0.05 for all tests of the whole study. Accuracy errors were calculated as the root mean square (RMS) of the individual relative differences between the directly measured fat volumes (Archimedes method) and the MR-based measurements of the ten different phantoms. Differences between accuracy errors were evaluated by a two-sided Student´s t-test for related samples, as the Kolmogorov-Smirnov-analysis revealed no significant difference from a normal distribution for all measurements and errors. Additionally, Pearson correlation coefficients of the MR measurements versus the directly measured fat volumes were calculated. Significant differences in correlation coefficients were assessed using the Fisher-Z-Transformation. Reproducibility errors were calculated as the root mean square of the individual coefficients of variation of the repeated measurements in each infant. The coefficients of variation were determined as the standard deviation of the repeated measurements in one infant divided by the mean of the measurements in that infant. Differences between reproducibility errors were evaluated by a two-sided Student´s t-test for different samples. The statistical computations were processed using SPSS Version 15.0 (Chicago, IL, USA).

## Results

### Phantom studies

**Fig 1:**
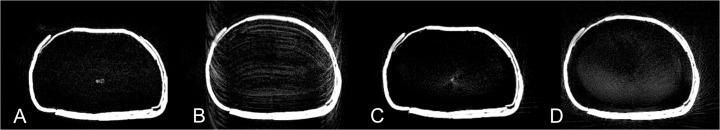
The phantom scanned by MR imaging with the cartesian wsTSE sequence without (A) and with breathing simulation (B) and the PROPELLER wsTSE sequence without (C) and with breathing simulation (D).

As determined with the Archimedes method, the average fat volume of the ten phantoms was 172 ± 72ml (range 54–299ml). In case of the MR measurements, the fat volume was over-estimated by the k-means clustering segmentation for all sequences (Fig. 1, Table 2) and underestimated by the threshold-based segmentation for all sequences. This error was minimal for the threshold 150, but pronounced for 250 and 350.

**Table 2:** *Absolute values* of the *in-vitro* phantom measurements determined with the different sequences (prop: PROPELLER TSE; cart: cartesian TSE) and segmentation algorithms (k-means: k-means clustering; thr150–350: threshold-based with different threshold settings of 150, 250 and 350).

	motionless		simulated breathing	
Segmentation	prop	cart	prop	cart
k-means [ml]	180 ± 82	190 ± 88	177 ± 81	180 ± 82
thr150 [ml]	163 ± 75	147 ± 64	162 ± 67	165 ± 74
thr250 [ml]	103 ± 44	116 ± 51	92 ± 46	106 ± 46
thr350 [ml]	60 ± 31	84 ± 47	49 ± 32	73 ± 41

The phantoms had an average volume of 172 ± 72 ml as determined with the Archimedes method.

Smallest accuracy errors were found for the conventional TSE sequence with k-means clustering segmentation (5.4%) in the motionless setting (Table 3). When simulating breathing artifacts, errors increased for both sequences and all segmentation procedures; however this was significant only in case of the threshold-based segmentation and the PROPELLER TSE sequence. Smallest errors in case of simulated breathing were found for the conventional TSE sequence with threshold-based segmentation (thr1, 9.1%). However, differences between k-means clustering and threshold-based segmentation with the smallest threshold (150) were not significant. Accuracy errors significantly increased when choosing higher thresholds (250, 350), up 76% in case of an inappropriate threshold (Table 3). Breathing artifacts increased the minimal error to 9%, while the chosen sequence did not significantly influence accuracy, although accuracy errors were slightly smaller in case of the conventional TSE sequence (Table 3). MR measures were highly correlated with the standard of reference (up to r^2^ = 0.996, Table 3). As in case of the accuracy errors, no difference was found between sequences or segmentation methods, if an appropriate threshold was used.

**Table 3:** *In-vitro accuracy errors* of the different sequences (prop: PROPELLER TSE; cart: cartesian TSE) and segmentation algorithms (k-means: k-means clustering; thr150–350: threshold-based with different threshold settings of 150, 250 and 350) expressed as root-mean-squares of the relative difference of the MR measurements versus real fat volume and Pearson correlation coefficients of the MR measurements versus real fat volume.

		motionless		simulated breathing	
	Segmentation	prop	cart	prop	cart
Relative difference (RMS)	k-means	7.4%	5.4%	14.7%	10.3%
	thr150	8.5%^†^	8.0%	15.3%* ^†^	9.1%*
	thr250	41.3%^†^	36.4%	52.0%* ^†^	41.3%*
	thr350	57.7%* ^†^	34.0%* ^†^	75.7%* ^†^	62.8%* ^†^
Pearson r^2^	k-means	0.986	0.996	0.966	0.969
	thr150	0.980	0.968	0.974	0.944
	thr250	0.871	0.922	0.918	0.803
	thr350	0.808	0.928	0.892	0.986

* indicates a significant (P<0.05) difference between prop and cart sequences,

^†^ between motionless sequences and sequences with breathing simulation.

### Infant studies

**Fig 2:**
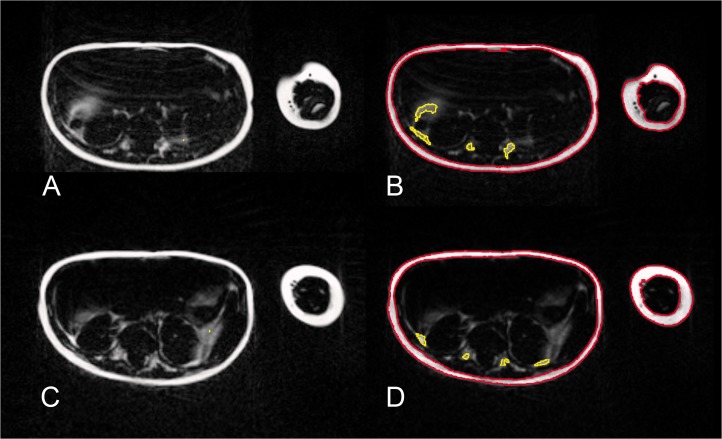
Infant scans with the cartesian wsTSE sequence (A,B) and the PROPELLER wsTSE sequence (C,D) and the corresponding threshold-based segmentations using a threshold of 150.

**Fig 3:**
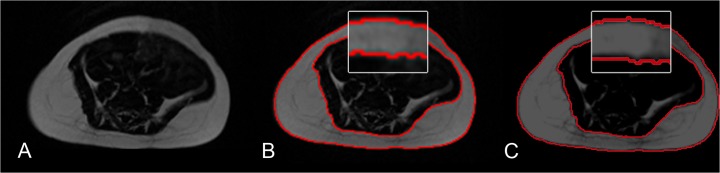
Infant scans with the cartesian wsTSE sequence (A), the k-means clustering segmentation of subcutaneous fat (B), and one threshold-based segmentation of subcutaneous fat using a threshold of 150 (C). The k-means clustering segmentation better matches the visual findings (inserts).

The average total fat volume was 1988ml in the 4 infants measured by the PROPELLER wsTSE sequence and evaluated with the k-means clustering segmentation (Table 4). The 7 infants measured by the conventional wsTSE sequence had a higher mean age (77 vs. 54 days) and a mean fat volume of 2233ml. The highest reproducibility error for total adipose tissue was 3.4% (Table 5). No significant differences were found between sequences or segmentation algorithms (Fig. 2), however smallest values were found for the k-means clustering segmentation technique and the PROPELLER wsTSE sequence (2.6%, p>0.05, Table 5, Fig. 3). Reproducibility errors significantly increased if subcutaneous (s.c.) and intra-abdominal (i.a.) fat depots were separated (Table 5).

**Table 4:** *Absolute values* of the whole body fat measurements in the ten infants of the different sequences (prop: PROPELLER TSE; cart: cartesian TSE) and segmentation algorithms (k-means: k-means clustering; thr150, thr250: threshold-based with different threshold settings of 150 and 250)) for whole body adipose tissue (total), subcutaneous and intra-abdominal adipose tissue.

	total		subcutaneous		intra-abdominal	
Segmentation	prop	cart	prop	cart	prop	cart
k-means [ml]	1988 ± 402	2233 ± 432	1605 ± 446	1834 ± 361	449 ± 33	256 ± 65
thr150 [ml]	1820 ± 368	2014 ± 339				
thr250 [ml]	1309 ± 420	1715 ± 438				

The separation in intra-abdominal and subcutaneous fat was only possible with the k-means clustering segmentation algorithm.

**Table 5:** *Reproducibility errors* for different sequences (prop: PROPELLER wsTSE, n(infants) = 4, n(scans) = 13; cart: cartesian wsTSE, n(infants) = 7, n(scans) = 21) and segmentation algorithms (k-means: k-means clustering; thr150, thr250: threshold-based with different threshold settings of 150 and 250) for whole body adipose tissue (total), subcutaneous and intra-abdominal adipose tissue.

	total		subcutaneous		intra-abdominal	
Segmentation	prop	cart	prop	cart	prop	cart
k-means	2.6%	2.9%	3.4%	4.4%	18.2%	25.8%
thr150	2.9%	3.4%				
thr250	2.6%	2.9%				

The separation in intra-abdominal and subcutaneous fat was only possible with the k-means clustering segmentation algorithm.

## Discussion

This study demonstrated that assessment of adipose tissue of young infants by whole body MRI is challenging and might not be possible in every infant without sedation. However, if the infants fall asleep, fat quantification is possible by MRI with high accuracy and reproducibility. While no significant difference was found between cartesian and PROPELLER wsTSE sequences, an adequate segmentation technique is mandatory for high accuracy. HASTE and gradient echo sequences were not feasible due to the high and inconsistent noise level.

To our knowledge, this is the first study to report accuracy for fat measurements in infant-like phantoms and reproducibility for total body fat measurements in young infants based on whole-body MR imaging. Harrington et al. reported intra- and inter-observer variability of up to 2.4% and 7%, respectively, in newborn infants [11]. Our values are similar, although Harrington et al. only assessed the reproducibility of the segmentation, as MR scans were not repeated and thus possible errors of bowel movement or breathing artifacts were not evaluated. In this regard, also the age of the infants matters, as the infants need to fall asleep for good image quality. No previous study reported scans of infants aged between 6 weeks and 6 months [8,11,19,20]; in our experience, newborns settle much easier as compared to the age group of infants participating in our study. Kullberg et al calculated reproducibility for automated fat measurements in adults and found single errors of up to 30% in case of visceral adipose tissue[21]. They did not calculate a root-mean-square error as recommended by Gluer et al. for quantitative techniques, thus their results are not comparable with other studies [22]. Ludescher et al. compared MR fat measurements with anthropometric measures in adults and stated that visceral adipose tissue can only be measured with cross sectional imaging techniques [23].

In infants and children, MRI may be an attractive tool to assess fat distribution as it lacks ionizing radiation. While Kullberg found no significant difference between CT and MRI [24], Yoon et al. reported intra- and interobserver reproducibilities of 15% to 22% in case of MRI in adipose adults, compared to <1% in CT [25]. This demonstrates the necessity of techniques that compensate for bowel and breathing motion artifacts occurring in MRI, in particular in infants, where breath-holding techniques are not feasible. An MR sequence with an integrated navigator pulse may be used, but the breathing frequency in young infants is too fast to use this technique in spin echo sequences. We used a propeller sequence for this purpose and visible artifacts were substantially reduced, as previously reported in other settings [10,26,27]. Also reproducibility errors were smaller in case of the PROPELLER sequence, however, differences were not significant. In contrast, in the phantom studies accuracy errors were slightly smaller for the conventional sequence both with and without simulated breathing. This may be explained by the slightly higher spatial resolution of the conventional sequence.

**Fig 4:**
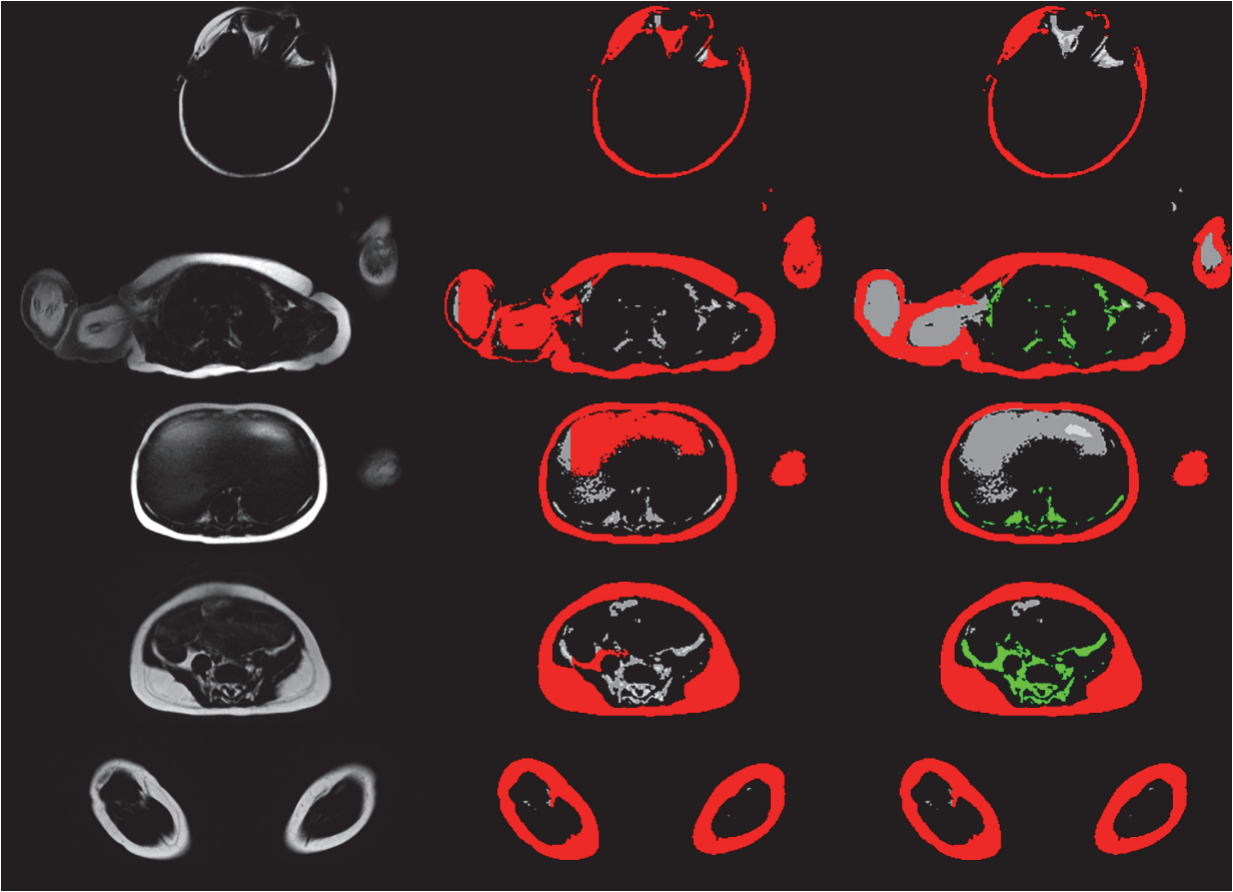
Five representative slices of one infant. Left row: cartesian wsTSE sequence; middle row: original segmentation using the k-means clustering algorithm; right row: manually corrected segmentation with separation of internal (green) and external fat (red). This scan represents a case with insufficient water suppression at the arms, where the most user interaction among all scanned infants was required. The complete scan is available as (S1 Fig.).

We also tried to use other sequences like a HASTE sequence, that is less sensitive to motion, however all infants awoke during the scan due to the inconsistent noise pattern and no valid data was acquired. Most studies used T1-w sequences for fat quantification as there is good image contrast between fat and other tissues [8,11,21,23–25]. In contrast, Peng et al. proposed water-saturated magnetic resonance imaging for accurate abdominal fat quantification, as otherwise signals from other tissues might influence segmentation in partial volume voxels [17]. This error is dependent on the surface-to-volume ratio of the adipose tissue and thus may play a significant role in particular in infants. We also used a water-saturated fast spin echo sequence, yielding high accuracy and reproducibility. However, there are also drawbacks of this technique, as water saturation might not be perfect in extremities due to local inhomogeneities of the magnetic field. A pure automatic segmentation was not possible in these cases (Fig. 4, S1 Fig.). Another problem is the separation of subcutaneous and internal fat. In young infants as investigated in this study, just a small amount of intra-abdominal fat is present at all, most of it retro- and not intraperitoneal [28,29]. Bowel motion created additional artifacts and the fatty milk in bowels and stomach could not sufficiently be suppressed by the water-saturated sequences. This was reflected by the high reproducibility errors of up to 26% in case of internal fat and made the sole quantification of intraperitoneal fat impossible. Of note, quantification of intraperitoneal fat only has not been reported in young infants up to now [8,11,19,20].

Many different segmentation techniques have been used and compared in adults so far [12,18,30,31]. Semi-automatic algorithms showed clear advantages compared to a manual segmentation [30]. We also compared two semi-automatic algorithms. Compared to adults, fat tissue in infants has a much higher surface-to-volume ratio leading to more pronounced errors due to incorrect surface-delineation. In our study, an automated surface-delineation by k-means-clustering showed higher accuracy as compared to a threshold-based method. Of note, these errors cannot be detected in reproducibility measurements that usually are performed for comparison of different algorithms or software packages in adults [30]. Compared to our study, similar algorithms have been presented in adults before [18,31], but phantom measurements, showing the difference of a cluster-based to a threshold-based approach, are presented here for the first time.

A few limitations have to be considered in this study. First, long-term reproducibility might be higher and was not assessed in this study. Second, we did not calculate inter-observer variability; however, the semi-automatic segmentation only required minimal corrections in most cases, thus observer influence is thought to be minimal in this setting. Third, we did not simulate bowel movement in the phantom measurements, thus no data about accuracy for intra-abdominal fat quantification is available.

In conclusion, assessment of whole body fat tissue was possible with very good reproducibility in young infants by whole body MR imaging; phantom studies revealed high accuracy in measuring subcutaneous fat, while the quantification of the intra-abdominal fat only remains difficult.

## Supporting Information

S1 Fig Every second slice of one 3D data set of one infant. Left row: cartesian wsTSE sequence; middle row: original segmentation using the k-means clustering algorithm; right row: corrected segmentation with separation of internal (green) and external fat (red). This scan represents a case with insufficient water suppression at the arms, where the most user interaction among all scanned infants was required.
(TIF)
