# Supplementary figures and images for: Accuracy and Reproducibility of Adipose Tissue Measurements in Young Infants by Whole Body Magnetic Resonance Imaging
Source: PLoS One. 2015 Feb 23;10(2):e0117127. doi: 10.1371/journal.pone.0117127 (PMC4338239; doi:10.1371/journal.pone.0117127)

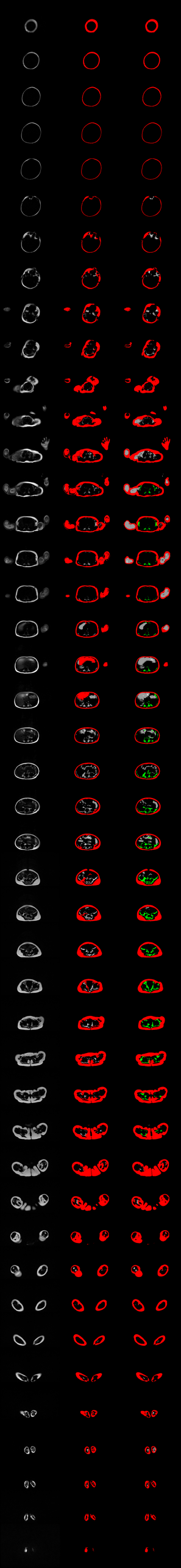

Supplement: S1 Fig — Left row: cartesian wsTSE sequence; middle row: original segmentation using the k-means clustering algorithm; right row: corrected segmentation with separation of internal (green) and external fat (red). This scan represents a case with insufficient water suppression at the arms, where the most user interaction among all scanned infants was required. (TIF) [file pone.0117127.s001.tif]
